# Misexpression of a transcriptional repressor candidate provides a molecular mechanism for the suppression of awns by *Tipped 1* in wheat

**DOI:** 10.1093/jxb/eraa106

**Published:** 2020-02-27

**Authors:** Tobias Würschum, Felix Jähne, Andrew L Phillips, Simon M Langer, C Friedrich H Longin, Matthew R Tucker, Willmar L Leiser

**Affiliations:** 1 State Plant Breeding Institute, University of Hohenheim, Stuttgart, Germany; 2 Rothamsted Research, Harpenden, UK; 3 School of Agriculture, Food and Wine, University of Adelaide, Urrbrae, SA, Australia; 4 CSIRO Agriculture and Food, Australia

**Keywords:** Association mapping, awns, *B1*, misexpression, *Tipped 1*, *Triticum aestivum*, wheat

## Abstract

Awns are bristle-like structures formed at the tip of the lemma on the florets of some cereal grasses. Wild-type wheat is awned, but awnletted and awnless variants have been selected and nowadays all forms are cultivated. In this study, we dissected the genetic control underlying variation of this characteristic feature by association mapping in a large panel of 1110 winter wheat cultivars of worldwide origin. We identified the *B1* (*Tipped 1*) locus on chromosome 5A as the major determinant of awnlessness globally. Using a combination of fine-mapping and expression analysis, we identified a putative C2H2 zinc finger protein with an EAR domain, characteristic of transcriptional repressors, as a likely candidate for *Tipped 1*. This gene was found to be up-regulated in awnless *B1* compared with awned *b1* plants, indicating that misexpression of this transcriptional regulator may contribute to the reduction of awn length in *B1* plants. Taken together, our study provides an entry point towards a better molecular understanding of the evolution of morphological features in cereals through selection and breeding.

## Teaser

We identified a candidate for the awnedness inhibitor *Tipped 1*, and showed that misexpression of this transcriptional repressor is the likely molecular cause underlying the evolution of awnlessness in wheat.

## Introduction

**Fig. 1.**
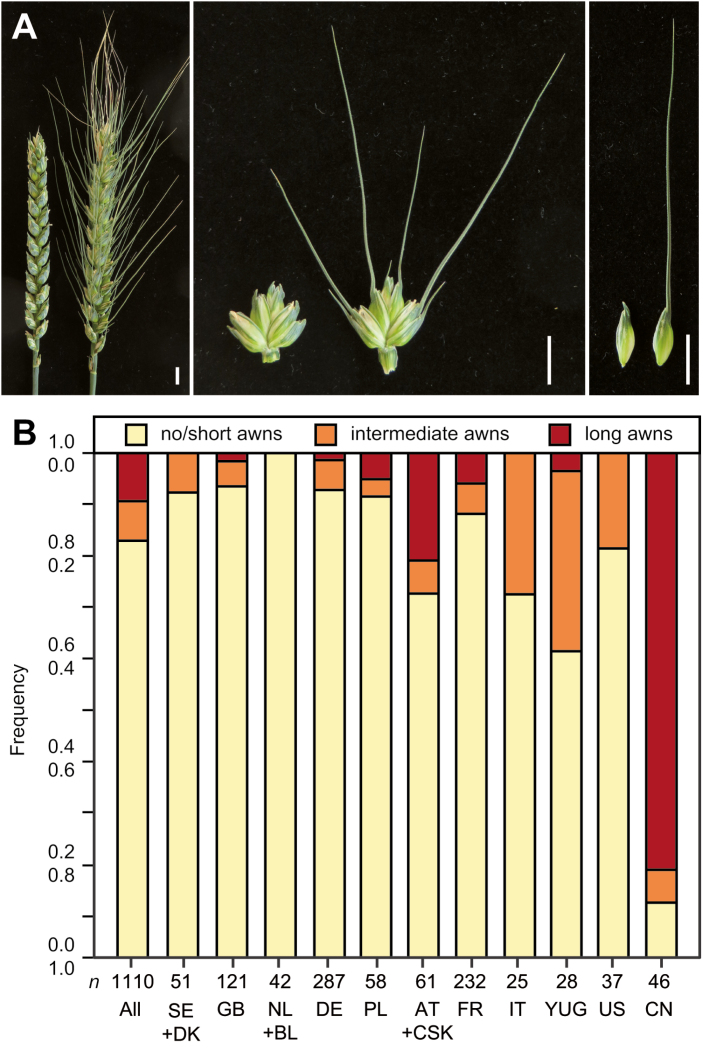
Awnedness in wheat. (A) Examples of an awnless and an awned wheat spike, floret, and lemma. The scale bar represents 1 cm. (B) Frequency of awnedness in the panel of 1110 winter wheat cultivars, scored as no/short awns (awns 0–4 mm), intermediate long awns (awns 5–25 mm), and long awns (awns>25 mm), dependent on the cultivars’ country of origin. AT, Austria; BE, Belgium; CN, China; CSK, former Czechoslovakia; DE, Germany; DK, Denmark; FR, France; GB, Great Britain; IT, Italy; NL, The Netherlands; PL, Poland; SE, Sweden; US, USA; YUG, former Yugoslavia, Serbia, Croatia.

Awns are stiff, bristle-like appendages characteristic of some grasses (*Poaceae*) such as wheat, barley, and rice, where they extend from the lemma of the florets (Fig. 1A). Wild-type wheat (*Triticum aestivum* L.) is awned, as are its progenitors and wild forms, while cultivated wheat incorporates awned, awnletted, and awnless forms. In the wild, awns aid in the dispersal of seeds to the site of germination, either by attaching seeds to passing animals, or by balancing the seed dispersal unit as it falls and propelling it into the ground (Harper *et al.*, 1970; Elbaum *et al.*, 2007). Awns can also provide protection from feeding damage by herbivorous animals, which conversely makes them an undesirable characteristic in forage crops (Christensen *et al.*, 1977; Karren *et al.*, 1994; Wallsten *et al.*, 2008). Furthermore, awns have been reported to contribute to carbohydrate storage, water-use efficiency, and photosynthesis, the latter hypothesized to be of particular importance after leaf senescence due to drought or diseases (e.g. Weyhrich *et al.*, 1995; Mozto and Giunta, 2002; Tambussi *et al.*, 2007).

Consequently, awned wheats should be the preferred type, but in many global wheat production areas awnless types predominate. It is often stated that awned wheats are better suited to warmer, drier environments while awnless wheats perform better in cooler, temperate regions (Teich, 1982; Rebetzke *et al.*, 2016). However, despite a considerable body of existing studies, the debate around the benefits and costs of awns for grain yield is ongoing and their effects probably depend on environmental conditions. Rebetzke *et al.* (2016) reported an overall equivalent yield of awned–awnletted sister near isogenic lines, but also showed that awns modify spike morphology, for example increasing grain size but decreasing fertile spikelet number. Based on this, Guo and Schnurbusch (2016) summarized the influence of wheat awns on grain yield as a balance between the costs of awn formation and the benefits of their various functions. The variation in awn length across wheat cultivars may therefore be due to some of their properties and the adaptation of wheat to the different target environments, but may also be a simple consequence of breeders’ preferences. Notably, short or non-existent awns also facilitate harvesting, handling, and storage of grains.

From a developmental genetic perspective, awns provide an interesting model to study the molecular mechanisms underlying the formation of morphological characteristics in grasses, but also the evolution of traits through domestication and breeding. In wheat, three major inhibitors of awn development are known: *Tipped 1* (*B1*), *Tipped 2* (*B2*), and *Hooded* (*Hd*), which are located on chromosomes 5A, 6B, and 4A, respectively (Watkins and Ellerton, 1940; McIntosh *et al.*, 2013). Genotypes homozygous for the wild-type *b1 b2 hd* alleles are fully awned, and as the awn length-reducing alleles act dominantly they have been designated *B1*, *B2*, and *Hd*. The two types of tip-awned wheats were designated *Tipped 1* and *Tipped 2*, which differ in the distribution of awn length along the spikes. *Tipped 1* produces very short awns at the base and center of the spike, but longer ones towards the apex, while for *Tipped 2* the awn tips are of nearly equal length along the spike, with slightly longer awns, if they present at all, at the center (Watkins and Ellerton, 1940). *Hooded* also reduces awn length, but leads to curved and deformed awns, often with membranous lateral outgrowth. Importantly, each of these alleles alone will only reduce awn length, while awnletted or awnless phenotypes result from combinations of at least two of these three inhibitors. *B1* or *B2* with *Hd* results in very short awns, *B1* with *B2* in very short or no awns, and the triple combination *B1*, *B2*, and *Hd* appears to be required for complete and stable awnlessness (Yoshioka *et al.*, 2017). *B1* has been fine-mapped to a 7.5 cM interval (Mackay *et al.*, 2014), and only recently was a putative candidate gene reported that may underlie the locus (DeWitt *et al.*, 2020; Huang *et al.*, 2020).

In this study, we phenotyped awns in a large panel of 1110 winter wheat cultivars of worldwide origin. We identified one major quantitative trait locus (QTL) on chromosome 5A, corresponding to *Tipped 1* (*B1*), explaining 75% of the genotypic variation. The two alleles at this locus separated awned from awnletted or awnless cultivars. Fine-mapping and expression analysis led to the identification of a putative zinc finger transcription factor, containing an EAR motif characteristic of transcriptional repressors, as the candidate underlying *B1*. Although no polymorphism was found in the gene itself, a stronger expression signal was detected in *B1* genotypes with reduced awn length compared with fully awned wild-type *b1* plants. Collectively, our results substantiate the findings from the two recent publications (DeWitt *et al.*, 2020; Huang *et al.*, 2020) and suggest misexpression of *B1* to be a likely molecular mechanism underlying the reduction of awn length by *Tipped 1* in wheat.

## Materials and methods

### Plant material and experimental design

This study is based on a panel of 1110 soft winter wheat (*T. aestivum* L.) cultivars that has been described in detail previously ( Würschum et al., 2015, 2017; Boeven *et al.*, 2016). In brief, it includes cultivars from worldwide origin released during the past decades, but with a focus on cultivars from Europe. Principal coordinate and phylogenetic analyses based on genome-wide markers revealed no major population structure (Boeven *et al.*, 2016). The test locations were Hohenheim [HOH, 48°42'54.4''N, 9°11' 22.6''E, 400 m above sea level (asl)] where 460 genotypes were evaluated in 2012, and in 2013 the entire panel was evaluated in Hohenheim, at Ihinger Hof (IHO, 48°44'42.6''N, 8°55'30.8''E, 493 m asl), and in Eckartsweier (EWE, 48°3'18.3''N, 7°52'16.8''E, 141 m asl). The experiment was conducted in a partially replicated design with a replication rate of 1.25 per location (Williams *et al.*, 2011). Entries were sown in observation plots of two rows and 1.25 m length.

Awns were scored on a scale from 0 to 2, where 0 denotes no or very short awns with a length between 0 mm and 4 mm, 1 denotes awns with a length between 5 mm and 25 mm, and 2 indicates awns with a length >25 mm. Phenotypic data were analyzed as described previously (Würschum *et al.*, 2017). In brief, best linear unbiased estimates (BLUEs) were estimated across all four environments, assuming fixed effects for the genotype. Heritability (*h*^2^) was estimated following the approach suggested by Piepho and Möhring (2007; formula [19]). All statistical analyses were performed using the statistical software R (R Development Core Team, 2014) and ASReml-R 3.0 (Gilmour *et al.*, 2009).

### Genotypic analysis and association mapping

All lines were genotyped by genotyping-by-sequencing (GBS) at Diversity Arrays Technology (Yarralumla, Australia) using the Wheat GBS 1.0 assay (DArTseq), resulting in silico DArT markers that show the presence or absence of DArT clones and single nucleotide polymorphism (SNP) markers present in some DArT clones. Markers with a minor allele frequency <0.05 were removed, resulting in a total of 23 720 markers for which a map position was available (Li *et al.*, 2015). The CloneIDs of the silico DArT markers were given a ‘D’ prefix and those of the SNP markers a ‘S’ prefix. Marker imputation was done with LinkImpute (Money *et al.*, 2015). The physical positions of the markers were determined by BLASTing the sequences of the DArT clones against the wheat genome (IWGSC RefSeq v1.0).

For association mapping, an additive genetic model was chosen and mapping was done with a mixed model incorporating a kinship matrix as described previously (Würschum *et al.*, 2017). To control for multiple testing, a Bonferroni-corrected threshold of *P*<0.01 was applied. The total proportion of genotypic variance (*p*_*G*_) explained by the detected QTL was calculated by fitting the significantly associated markers in the order of the strength of their association simultaneously in a linear model. The ratio *p*_*G*_=R2adj / *h*^*2*^, where R2adj refers to the adjusted R2 from the linear model and *h*^*2*^ to the heritability of the trait, yielded the proportion of genotypic variance (Utz *et al.*, 2000). The *p*_*G*_ values of individual QTL were accordingly derived from the sums of squares of the QTL (SS_QTL_) in this linear model. The allele substitution (α) effects were derived as the regression coefficient from models with only the marker under consideration.

### Expression analysis

To study the expression of the three candidate genes, TraesCS5A01G542600, TraesCS5A01G542700, and TraesCS5A01G542800, spikes of awned and awnless genotypes were sampled in the field at the stage when the second node of the stem is visible (BBCH stage 32). RNA was extracted with the Qiagen RNeasy^®^ Plant Kit (QIAGEN GmbH, Hilden, Germany) and reverse transcribed into cDNA with the M-MuLV reverse transcriptase from Genaxxon bioscience (Ulm, Germany). The sequences of the three genes were BLASTed against the wheat genome to identify genes with sequence similarity, which for all three identified homoeologous genes on chromosomes 4B and 4D. The obtained sequences were aligned to generate gene-specific primers for the quantitative PCR (qPCR).

For TraesCS5A01G542600, the primers were 5'-TTGTGATCGGGCAGACGTTT-3' as forward primer and 5'-GGCTCCTGCACCAGTTTGT-3' as reverse primer, and the PCR was run at an annealing temperature of 63 °C. For TraesCS5A01G542700, the primers were 5'- GGCTGGAGAAGCTCCTTGTG-3' as forward primer and 5'- GTTGAGATGGCCCTTGTATACAGTC-3' as reverse primer, and the PCR was run at an annealing temperature of 60 °C. For TraesCS5A01G542800, the primers were 5'-CCACCAGAACGCTCACAAGCT-3' as forward primer and 5'-ACGATATCCTGCTCGCCAAGC-3' as reverse primer, and the PCR was run at an annealing temperature of 65 °C. As the control gene, we used Ta2291 (ADP-ribosylation factor) (Paolacci *et al.*, 2009). qPCR was performed on a Roche LightCycler^®^ 480 II with the Genaxxon bioscience GreenMasterMix and two (TraesCS5A01G542600, TraesCS5A01G542700) or four (TraesCS5A01G542800) technical replications per genotype.

## Results

### Variation of awnedness in wheat

We investigated awnedness in a panel of 1110 winter wheat cultivars of global origin grown in field trials at four environments (Fig. 1A). Awnedness was classified into three categories: no or short awns (awns 0–4 mm), intermediate long awns (5–25 mm), and long awns (>25 mm). We observed a significant genotypic variance and a comparably low genotype-by-environment interaction variance, indicating that awnedness is mainly determined by the genotype (see Supplementary Table S1 at *JXB* online). Consequently, the heritability was high at 0.94. Most of the cultivars of this panel had no or short awns (82.6%), some had intermediate long awns (7.8%), and 9.6% had long awns (Fig. 1B). This can mainly be attributed to the composition of the panel, with a majority of the cultivars originating in Europe, where the awnless types are predominant. For example, of the British, French, and German cultivars, only 1.7, 6.0, and 1.4%, respectively, had long awns. The highest frequency (82.6%) of long awns was found in the Chinese lines. In general, there appeared to be a higher frequency of the intermediate or long-awned genotypes in the countries of lower latitude, which might indicate an adaptive advantage under the warmer and drier conditions prevalent in these regions. Alternatively, this might be due to breeders’ preferences or the composition of this panel. Nevertheless, the available variation presented a good entry point to study the genetic control underlying this trait.

### Identification of *Tipped 1* as the major QTL underlying awnedness

**Fig. 2.**
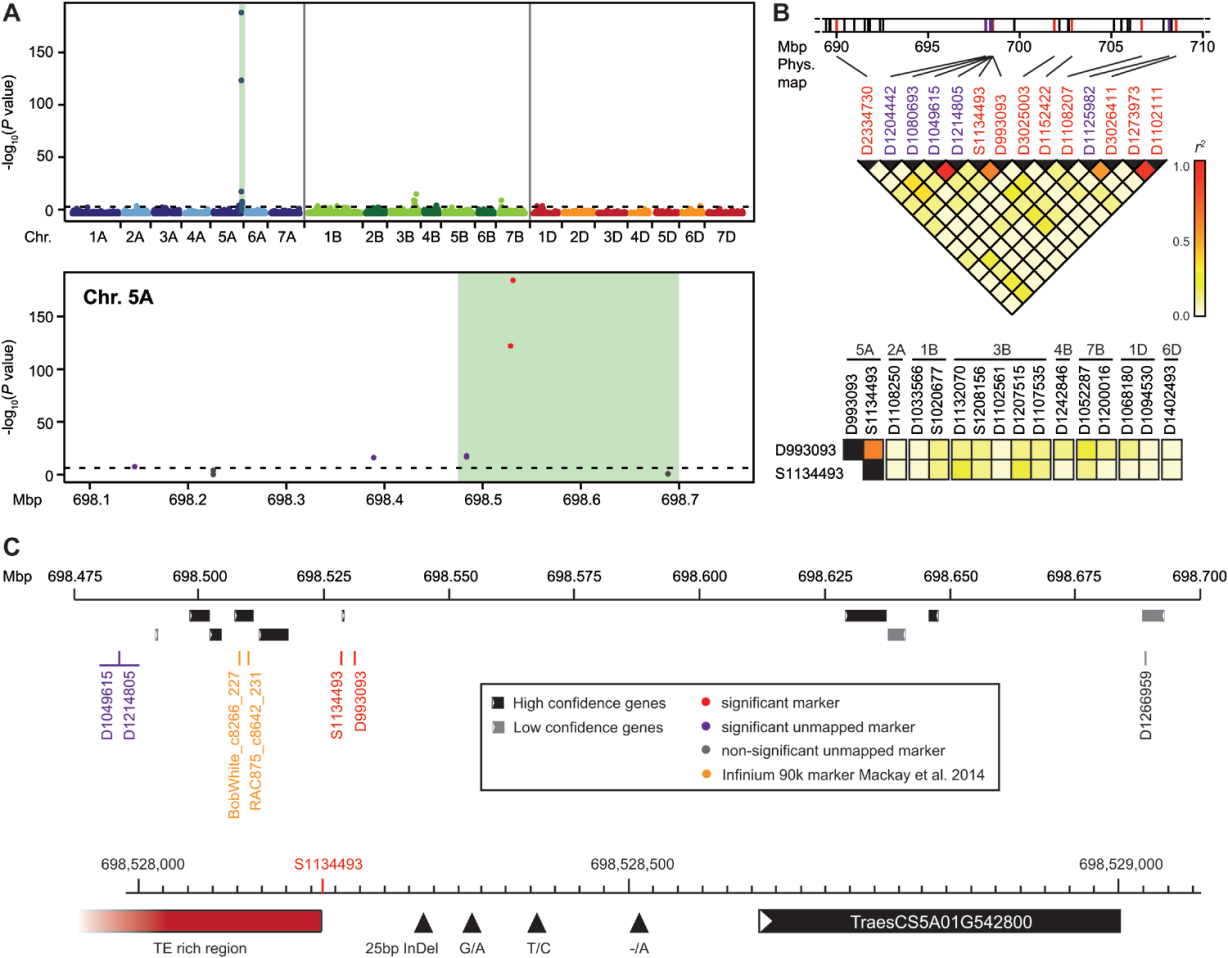
Fine-mapping of the major awnedness inhibitor locus *B1* (*Tipped 1*). (A) Manhattan plots showing results from the genome-wide association mapping and subsequent narrowing down of the target region. (B) Linkage disequilibrium structure at the *B1* (*Tipped 1*) locus on chromosome 5A and between the two most significantly associated markers of the *Tipped 1* locus (D993093 and S1134493) and markers of the other putative QTL. (C) Fine-mapping of the *Tipped 1* locus and identification of the candidate gene.

Genome-wide association mapping based on 23 720 mapped markers identified a major QTL at the end of the long arm of chromosome 5A (Fig. 2A, B). Additional putative QTL were found on several other chromosomes, most notably on 3B (Supplementary Table S2). In Chinese Spring, which is awnless and presumably *B2 Hd*, deletion of the short arm of chromosome 3B resulted in a partially awned phenotype, suggesting another awn development locus located on this chromosome (Ma *et al.*, 2012). Intriguingly, the five markers on chromosome 3B are genetically mapped to this chromosome, but their physical positions were found to be on chromosome 5A, in the region of the major QTL. The linkage disequilibrium (LD) between these markers and those indicative of the major QTL on 5A was rather low, indicating that they present a separate locus and were not identified due to their LD with the major QTL (Fig. 2B). However, owing to the very strong association of the 5A QTL, the latter cannot be ruled out completely.

Regarding the proportion of explained genotypic variance, one QTL dominates in this panel: the QTL on chromosome 5A explaining 74.6% (Supplementary Table S2). Owing to its chromosomal localization, we concluded that this locus corresponds to the major awnedness inhibitor locus *B1* (*Tipped 1*). It was identified by several significantly associated markers, of which two showed much stronger association signals than all the others (Fig. 2A). The proportion of explained genotypic variance can almost fully be captured by one marker, suggesting a single gene underlying this locus. Analysis of the LD among the significant markers indicated a rapid LD decay, which facilitates fine-mapping (Fig. 2B).

**Fig. 3.**
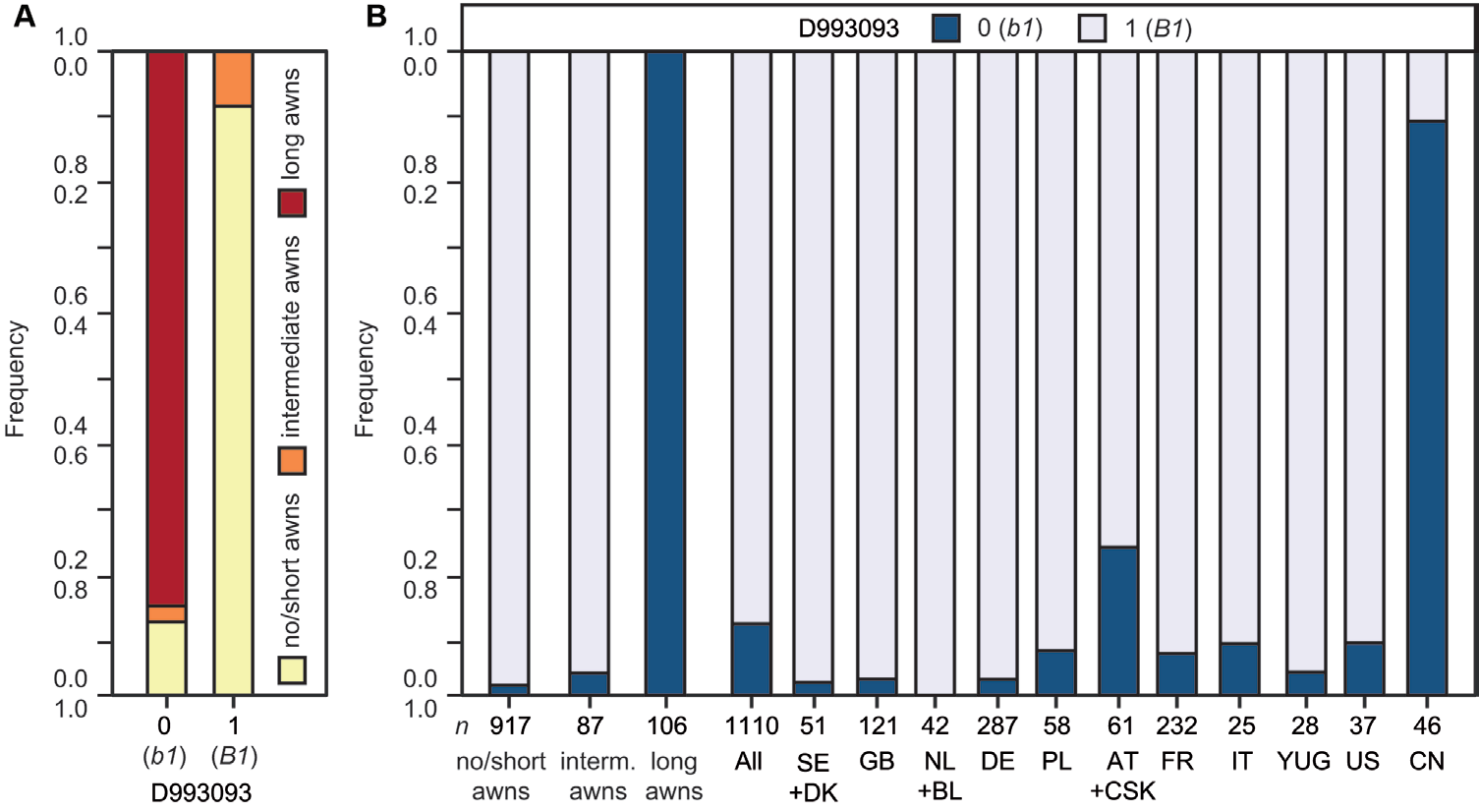
Effect of the *B1* (*Tipped 1*) locus on awnedness in wheat. (A) Awnedness dependent on *Tipped 1* (marker D993093) with the wild-type *b1* and the awnlessness-conferring *B1* alleles. (B) Frequency of *b1*/*B1* (D993993) in different countries of origin.

Using the most strongly associated marker D993093 (physical position at base pair 698 530 966) as a proxy for *Tipped 1*, we found that most of the wild-type *b1* plants had long awns (86.2%), few had intermediate long awns (2.4%) possibly due to either *B2* or *Hd*, and some had no or short awns (11.4%), possibly carrying *B2* and *Hd* (Fig. 3). Of the *B1* plants, 91.5% had no or short awns and the remainder had intermediate awns. Thus, all 106 cultivars with long awns carried the wild-type *b1*, while the vast majority of the genotypes with no, short, or intermediate long awns carried *B1*. The example of Chinese Spring illustrates that awnlessness can also be achieved through *B2* and *Hd*, possibly in combination with other QTL, but does not necessarily require *B1*. Our results substantiate, however, that *B1* is a major and widespread component of awnlessness in winter wheat, particularly for the European countries of origin, but probably even on a global scale (Mackay *et al.*, 2014; DeWitt *et al.*, 2020; Huang *et al.*, 2020).

### Fine-mapping of *Tipped 1*

We narrowed *Tipped 1* down to a region at ~698.5 Mbp on chromosome 5A, close to two strongly associated markers recently reported by Mackay *et al.* (2014) who investigated this trait in a wheat MAGIC population (Fig. 2C; Supplementary Fig. S1). Our two most strongly associated markers flank the candidate gene TraesCS5A01G542800 at 698.531 Mbp, and downstream there is no annotated gene for ~100 kb, followed by a non-significant marker. In the other direction, the association signal also drops off rapidly, with the next two markers being much more weakly associated.

We therefore assessed the five genes TraesCS5A01G542400 to TraesCS5A01G542800 for polymorphisms in genotypes carrying the wild-type *b1* allele or presumed to carry the *B1* inhibitor allele. No polymorphisms were found in TraesCS5A01G542400 and TraesCS5A01G542500, including their promoter regions. In contrast, TraesCS5A01G542600 and TraesCS5A01G542700 carry non-synonymous polymorphisms in the coding regions, as well as polymorphisms including InDels >10 bp in introns (Supplementary Fig. S2). No polymorphism was found in the coding region of TraesCS5A01G542800, but a 25 bp InDel was identified in the promoter at position –346 bp relative to the start codon, with awnless *B1* genotypes carrying the deletion. All polymorphisms formed one haplotype, i.e. followed the same pattern, with one allele in the *b1* genotypes and another in the presumed *B1* plants. TraesCS5A01G542600 is annotated as a sugar and other transporter, TraesCS5A01G542700 as a universal stress protein family gene with a protein kinase domain, and TraesCS5A01G542800 had no functional annotation. TraesCS5A01G542800 is a rather short gene, consisting of a single exon of 366 bp, and has two homoeologs on chromosomes 4D (TraesCS4D02G476700LC) and 4B (not annotated), of which the latter lacks the C-terminal region. A BLAST search against Arabidopsis, rice, and maize yielded several genes with sequence similarity, which revealed TraesCS5A01G542800 to be a C2H2 zinc finger, having the zinc finger domain as well as the EAR motif at the C-terminal end (Supplementary Fig. S3).

### Misexpression reveals a C2H2 zinc finger as the candidate for *Tipped 1*

For *Tipped 1*, the awned wild-type *b1* allele is recessive and the awn inhibitor *B1* allele is dominant. If the mutant version, *B1*, was a hypomorphic allele, *b1* would have to act as an activator of awn formation, in which case it is more likely that *b1* would be dominant or the phenotype intermediate with incomplete dominance, as dominance would require a threshold level of activity not reached in heterozygous plants (Supplementary Fig. S4). If, in contrast, *B1* was a hypermorphic allele, *b1* would have to be a repressor of awn formation, with an increase of this activity resulting in a dominant mode of action with complete repression of awns. This may be caused either by an allele with increased activity compared with the wild-type allele, or by a gene product with normal function, but produced at increased amounts. In addition, *B1* could be a neomorphic allele; that is, an allele that has gained a novel function not related to that of the wild-type allele or a novel expression pattern. While the former would be caused by mutations in the gene’s coding region, the latter would require changes in the regulatory region resulting in expression in tissues or developmental stages in which the gene is not normally expressed. Notably, mutant screenings of awnless cultivars have yielded awned mutants (Rakszegi *et al.*, 2010, Dhaliwal *et al.*, 2015), which is less likely for a hypomorphic allele that would have to restore its functionality, but is consistent with *B1* being a hypermorphic or a neomorphic allele. Mutations that disrupt the gene’s function would render the misexpressed transcript non-functional and reverse the phenotype, and, in the case of redundancy, without any other effect. Thus, misexpression, either overexpression or ectopic expression, appeared as a likely molecular cause for *B1*, and we therefore examined the expression of the candidate genes in wheat spikes.

**Fig. 4.**
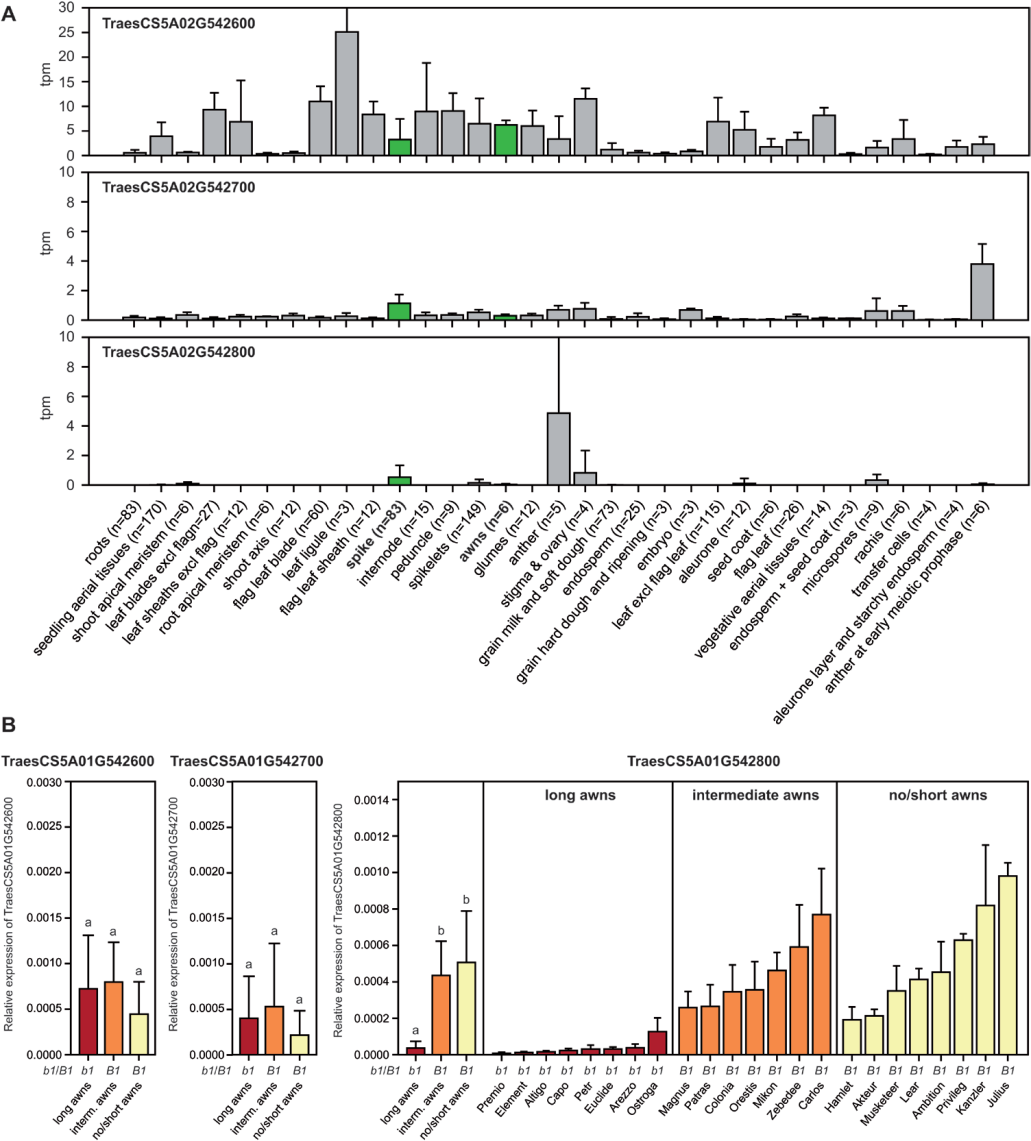
Expression analysis of candidate genes. (A) Expression of the three candidate genes TraesCS5A02G542600, TraesCS5A02G542700, and TraesCS5A02G542800 in different tissues. Data are taken from the Wheat Expression Browser expVIP (Ramírez-González *et al.*, 2018; Borrill *et al.*, 2016). (B) Expression of the three candidate genes in developing spikes of cultivars with long awns, intermediate long awns, or no/short awns. Samples were taken from at least 10 plants per genotype. The whiskers represent the SD and different letters indicate significant differences between the three groups. The presumed allelic state at *Tipped 1* (D993993) is indicated underneath.

Using available gene expression data in wheat, we found that all three candidate genes are expressed in spikes, as well as in awns (Fig. 4A). Both TraesCS5A01G542600 and TraesCS5A01G542700 appear to be more universally expressed, while TraesCS5A01G542800 showed a more specific expression, being restricted to tissues within spikes.

We next assessed the expression of the three candidate genes in developing, whole wheat spikes at around the stage when the second stem node is visible, using at least 10 plants per genotype. Expression was assessed in cultivars with no or short awns carrying *B1*, those with intermediate awns, and from wild-type *b1* plants with long awns. qPCR revealed no significant difference in expression for TraesCS5A01G542600 and TraesCS5A01G542700 (Fig. 4B; Supplementary Fig. S5). In contrast, for TraesCS5A01G542800, there was a significantly (*P*<0.01) higher expression in plants with no or short awns carrying *B1* compared with wild-type *b1* plants. Plants with intermediate long awns have been described as half-awned and considered to be allelomorphic with *B1* and *b1* [i.e. to carry another allele termed *b1*^*a*^ (Watkins and Ellerton, 1940)]. Cultivars with intermediate long awns were found to have expression levels similar to the short-awned genotypes, indicating that they carry the same allele. The different effects of this *B1* allele on awn length (debated previously) probably depend on the allelic state at *B2* and *Hd*, and potentially other QTL. Taken together, our results strongly suggest the C2H2 zinc finger protein TraesCS5A01G542800 as the gene underlying the major awnedness inhibitor *Tipped 1* in wheat, with misexpression of this gene resulting in a reduction of awn length.

This misexpression might be caused by the 25 bp InDel in the promotor region at position –346 relative to the start codon (Supplementary Fig. S2). Awnless *B1* genotypes such as Cadenza carry the deletion, which would mean that removal of this putative regulatory region results in overexpression or ectopic expression and, conversely, that the 25 bp region contains elements that restrict the expression of *Tipped 1* (Supplementary Table S3). Sequencing showed this InDel to coincide with the awn phenotype and the presumed *b1*/*B1* genotype in another 26 wheat cultivars (Supplementary Table S4). However, the haplotype structure with several linked polymorphisms does not allow us to conclude that this InDel is causative for the altered expression. Alternatively, another of the identified polymorphisms in the promoter region may cause the observed misexpression or another polymorphism in a regulatory region not assessed here. While further upstream is a region rich in transposable elements, delimiting what might classically be assumed as a promoter, the regulatory elements disrupted in *B1* might be further away from the gene itself, in either direction. For example, another 276 bp long InDel is located 575 bp downstream of the gene, again with Chinese Spring having the insertion and Cadenza the deletion. Notably, Huang *et al.* (2020) also identified the 25 bp InDel in the promoter region and reported it to be highly predictive of awn inhibition, whereas DeWitt *et al.* (2020) reported sequence variation in the promoter region to not be predictive for the phenotype but rather a 30 bp deletion ~4 kb downstream of the gene. Thus, further research is required to uncover the source behind the observed misexpression of *B1*.

## Discussion

Awns are a characteristic feature of many cereals and, in wheat, both awned and awnless cultivars exist. In rice, most wild species and most *Oryza sativa* ssp. *indica* lines produce awns, whereas in *O. sativa* ssp. *japonica* awns are partially or completely suppressed as a result of domestication and breeding (Toriba and Hirano, 2014). Genetic studies have indicated multiple genes to be involved in the formation and/or elongation of awns. *Awn-1* (*An-1*) is required for awn elongation and encodes a basic helix–loop–helix protein that regulates cell division and is strongly expressed at the apex of the lemma primordia (Luo *et al.*, 2013). The identification of additional genes suggested that different developmental processes are involved in awn formation (Toriba and Hirano, 2014). In barley, the dominant *Hooded* (*K*) replaces the awn by an extra flower of inverse polarity on the lemma. This homeotic transformation is caused by a 305 bp duplication in intron 4 of the homeobox gene *HvKnox3*, altering the strength and pattern of expression in the lemma (Müller *et al.*, 1995). The barley *short awn 2* (*lks2*) is a natural variant from Eastern Asia that shortens awns by about half and was found to encode a *SHORT INTERNODES* (*SHI*) family transcription factor (You *et al.*, 2012). However, the awn inhibitors of wheat do not appear to be orthologs of the awn development genes identified in rice and barley (Yoshioka *et al.*, 2017).

Using a combination of fine-mapping in a large diversity panel and expression analysis, we identified the C2H2 zinc finger TraesCS5A01G542800 as the likely candidate underlying the major awnedness inhibitor *Tipped 1* in wheat, the same candidate gene suggested by DeWitt *et al.* (2020) and Huang *et al.* (2020). It contains a zinc finger that enables binding to the target sequence and in the C-terminal region an ethylene-responsive element-binding factor (ERF)-associated amphiphilic repression (EAR) motif (Laity *et al.*, 2001). The EAR motif functions in active repression of transcription and is highly conserved in transcriptional regulators with known function as negative regulators across a range of developmental processes (Ohta *et al.*, 2001; Hiratsu *et al.*, 2002; Payne *et al.*, 2004; Kagale and Rozwadowski, 2011). Based on homology, it appears likely that the *B1* (*Tipped 1*) candidate TraesCS5A01G542800 functions as a transcriptional repressor.

While there was no polymorphism within the gene, we found increased expression of TraesCS5A01G542800 in spikes of *B1* plants compared with fully awned wild-type *b1* genotypes. This indicates that misexpression of this transcriptional repressor may contribute to *B1*, which is in line with its dominant nature. This misexpression might either directly or indirectly lead to the reduction of awn length and might be caused by polymorphisms in a regulatory region of the gene. Epigenetic modifications are another possible explanation, but appear less likely given the stability of the phenotype. Thus, while we have identified the misexpression of *B1*, its basis requires further research.

**Fig. 5.**
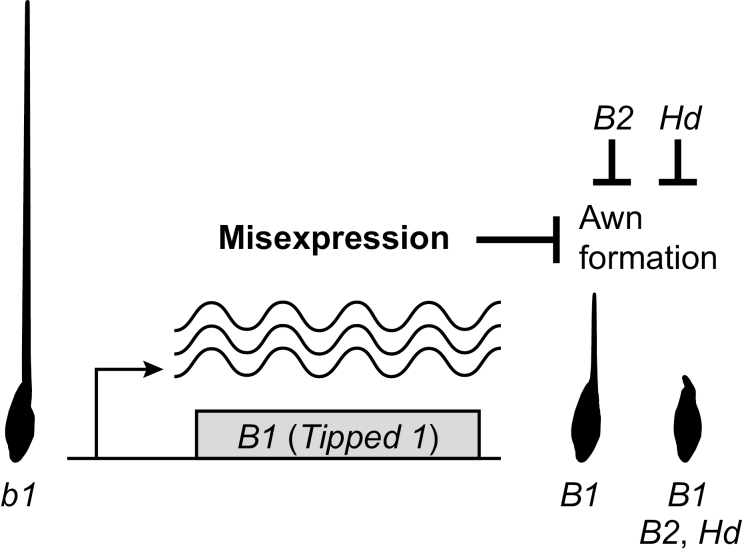
Model for the evolution of awnlessness in wheat by misexpression of *B1* (*Tipped 1*) resulting in a reduction of awn length. *B1* in combination with *B2* and/or *Hooded* (*Hd*) leads to awnless plants.

Our model suggests that through misexpression *B1* acts on downstream target(s), which reduces the activity of the awn development pathway to result in the formation of intermediate long awns (Fig. 5). This remaining activity may be due to an incomplete penetrance, in which case *B1* may be able to fully repress awns if expressed at sufficiently high levels. In combination with *B2* and/or *Hd*, this residual activity is further diminished, resulting in no or only short awns. *B2* and *Hd* may act in parallel to *B1*, but the epistatic interactions reported for these three awn inhibitor genes (Yoshioka *et al.*, 2017) rather suggest that they act in the same pathway or that their gene products act together. Interestingly, it has recently been shown that the TOPLESS family of co-repressors, that are involved in the regulation of development, hormone signaling, and stress response, interact with the EAR motif (Ke *et al.*, 2015). This, in combination with other results of genetic interactions, co-complex formation, and physical interaction, led to a model where EAR repressors coordinate development and environmental response by regulating expression of their targets via recruitment and action of chromatin-remodeling factors (Kagale and Rozwadowski, 2011).

Taken together, our results show that the awnedness inhibitor locus *B1* (*Tipped 1*) is widespread and probably the major determinant of awnlessness in wheat globally. We identified a C2H2 zinc finger protein with an EAR motif characteristic for transcriptional repressors as a candidate for *Tipped 1*, and show that its misexpression is consistent with the reduction of awn length in *B1* plants. Importantly, these findings lend further support to the results of two very recent studies that also identified the C2H2 zinc finger protein as a candidate underlying the *B1* locus. Further characterization of this gene and identification of the other known awnedness inhibitors *B2* and *Hd* will shed further light on the molecular mechanisms underlying the expression of this morphological characteristic in wheat.

## Supplementary data

Supplementary data are available at *JXB* online.

Fig. S1. Fine-mapping of *Tipped 1*.

Fig. S2. Polymorphisms in candidate genes.

Fig. S3. Alignment of protein sequences.

Fig. S4. Schematic presentation of *B1* function.

Fig. S5. Expression of candidate genes.

Table S1. Summary statistics for awnedness.

Table S2. Marker-explained variance.

Table S3. Analysis of the promotor InDel.

Table S4. Presence or absence of the InDel.

eraa106_suppl_supplementary_tables_S1-S4_figures_S1-S5
