# Supplementary material for: Misexpression of a transcriptional repressor candidate provides a molecular mechanism for the suppression of awns by Tipped 1 in wheat
Source: J Exp Bot. 2020 Feb 27;71(12):3428–36. doi: 10.1093/jxb/eraa106 (PMC7307850; doi:10.1093/jxb/eraa106)
Supplement: eraa106_suppl_supplementary_tables_S1-S4_figures_S1-S5 [file eraa106_suppl_supplementary_tables_s1-s4_figures_s1-s5.pdf]

Mis-expression of a transcriptional repressor candidate provides a molecular mechanism for the suppression of awns by *Tipped 1* in wheat

Tobias Würschum[1,†], Felix Jähne[1], Andrew L. Phillips[2], Simon M. Langer[1,*], C. Friedrich H. Longin[1], Matthew R. Tucker[3], Willmar L. Leiser[1]

[1] State Plant Breeding Institute, University of Hohenheim, 70593 Stuttgart, Germany

[2] Rothamsted Research, Harpenden AL5 2JQ, United Kingdom

[3] School of Agriculture, Food and Wine, University of Adelaide, Waite Campus, Urrbrae, SA, Australia

[*] Current address: BASF Agricultural Solutions Seed GmbH, Gatersleben, Germany

[†] Correspondence: tobias.wuerschum@uni-hohenheim.de

Supporting data

Table S1. Summary statistics for awnedness.

	Awns
Min	-0.20
Mean	0.31
Max	2.02
σ_G^2	0.350**
$\sigma_{G\times E}^2$	0.015**
σ_e^2	0.046
h^2	0.94

Genotypic variance (σ_G^2), genotype-by-environment interaction variance ($\sigma_{G\times E}^2$), error variance (σ_e^2), and heritability (h^2). ** significant at the 0.01 probability level.

Table S2. Proportion of explained genotypic variance of all mapped markers significant at the Bonferroni-corrected significance threshold of $P < 0.01$ in a joint fit of all the markers in the order of their P values (lowest modelled first).

Marker	Chr.	Pos. (cM)	Pos. (bp)[a]	P value	p_G[b]	α Effect	p[c]
D1108250	2A	137.6	579,695,033	8.3e-8	0.58	0.31	0.10
D2334730	5A	270.1	690,002,376	1.6e-8	0.50	-0.25	0.35
S1134493	5A	293.1	698,528,378	9.7e-123	1.83	-0.86	0.07
D3025003	5A	293.1	701,887,287	1.9e-9	0.04	-0.21	0.14
D993093	5A	293.6	698,530,966	3.4e-185	74.60	-0.81	0.11
D1152422	5A	294.3	702,849,155	8.7e-21	0.01	0.57	0.06
D3026411	5A	299.1	708,545,706	7.5e-12	0.07	0.26	0.20
D1273973	5A	299.1		4.2e-11	0.00	-0.06	0.64
D1108207	5A	299.1	706,661,604	5.8e-10	0.00	0.21	0.21
D1102111	5A	299.1		2.7e-9	0.03	-0.05	0.63
D1033566	1B	104.0	19,615,432	9.6e-8	0.06	-0.18	0.15
S1020677	1B	307.4	536,709,124	1.1e-7	0.63	0.54	0.27
D1132070	3B	247.7	5A: 708,449,614	1.2e-12	0.10	0.29	0.17
S1208156	3B	253.7	5A: 683,133,122	3.9e-10	0.01	0.39	0.05
D1102561	3B	260.3	5A: 706,549,464	4.8e-12	0.01	-0.05	0.62
D1207515	3B	272.2	5A: 708,449,611	9.7e-19	0.06	0.31	0.16
D1107535	3B	272.3	5A: 708,820,579	2.3e-7	0.02	0.20	0.28
D1242846	4B	129.9	653,711,185	2.9e-8	0.43	0.30	0.14
D1052287	7B	23.9	14,697,845	1.1e-12	0.51	-0.57	0.06
D1200016	7B	24.1		2.7e-7	0.13	0.24	0.20
D1068180	1D	43.6	12,986,193	1.4e-7	0.52	-0.41	0.11
D1094530	1D	98.1	49,977,861	9.3e-8	0.71	0.28	0.15
D1402493	6D	187.2	462,479,338	7.9e-8	1.10	0.25	0.24

[a] physical positions of the markers based on IWGSC RefSeq v1.0

[b] proportion of explained genotypic variance

[c] frequency of the allele increasing awnedness

Table S3. Analysis of the TraesCS5A01G542800 promotor InDel sequence for transcription factor binding sites by PlantPAN 3.0.

TraesCS5A01G542800 promotor InDel sequence:

TCACGAAATTC**CACGGAAGTAGGCCAGGTACAAATA**CACGGAAGTA

TCACGAAATTC--------------------------CACGGAAGTA

1 11 37 46

Arabidopsis thaliana:

Matrix ID	Family	Position	Strand	Similar Score	Hit Sequence	TF ID or Motif name
TFmatrixID_0605	B3	32	-	0.86	aaaTACACgg	AT4G31610
TF_motif_seq_0244	SBP	27	-	1	GGTAC	AT2G33810; AT2G47070
TF_motif_seq_0244	SBP	28	+	1	GTACA	AT2G33810; AT2G47070
TF_motif_seq_0257	NF-YB;NF-YA;NF-YC	31	+	0.8	CAAAT	AT1G09030; AT1G17590; AT1G21970; AT1G30500; AT1G54160; AT1G54830; AT1G56170; AT1G72830; AT2G38880; AT2G47810; AT3G05690; AT3G14020; AT3G20910; AT3G53340; AT4G14540; AT5G06510; AT5G12840; AT5G27910; AT5G38140; AT5G47640; AT5G47670; AT5G50470; AT5G50480
TF_motif_seq_0258	Dehydrin	19	-	0.8	GTAGG	U01377
TF_motif_seq_0267	Trihelix	27	+	0.8	GGTAC	AT5G01380
TF_motif_seq_0271	bZIP	1	+	0.8	TCACG	AT1G77920; AT3G12250; AT5G06950; AT5G06960; AT5G10030; AT5G65210; AT1G22070
TF_motif_seq_0508	SBP	25	+	0.75	caGGTACaa	AT1G20980; AT1G27360; AT1G27370; AT1G53160; AT1G69170; AT1G76580; AT2G33810; AT2G42200; AT2G47070; AT3G15270; AT3G57920; AT3G60030; AT5G18830; AT5G43270
TFmatrixID_1354	NAC; NAM	28	+	0.9	gtacaaatACACGgaa	AT5G07680
TFmatrixID_1563	NAC; NAM	9	-	0.91	ttccACGGAagta	AT1G52890
TFmatrixID_1565	NAC; NAM	9	+	0.89	ttcCACGGaagta	AT1G52890
TFmatrixID_1567	NAC; NAM	34	+	0.96	atACACGgaagt	AT1G52890
TFmatrixID_1571	NAC; NAM	9	+	0.92	ttcCACGGaagta	AT3G15500
TFmatrixID_1572	NAC; NAM	34	+	0.98	atACACGgaagt	AT3G15500
TFmatrixID_1573	NAC; NAM	9	+	0.89	ttcCACGGaagta	AT3G15500
TFmatrixID_1575	NAC; NAM	9	-	0.91	ttcCACGGaagta	AT3G15500
TFmatrixID_1576	NAC; NAM	9	+	0.91	ttcCACGGaagta	AT3G15500
TFmatrixID_1578	NAC; NAM	34	+	0.95	atACACGgaagt	AT3G15500
TFmatrixID_1584	NAC; NAM	34	+	0.86	atACACGgaagt	AT5G39610
TFmatrixID_1604	NAC; NAM	34	+	0.93	atACACGgaagt	AT1G01720
TFmatrixID_1659	NAC; NAM	35	+	0.93	tACACGgaagt	AT1G69490
TFmatrixID_1673	NAC; NAM	34	+	0.87	atACACGgaagt	AT3G49530
TFmatrixID_1703	NAC; NAM	10	+	0.9	tcCACGGaagta	AT5G66300
TFmatrixID_1706	NAC; NAM	34	+	0.96	ataCACGGaagt	AT5G66300
TFmatrixID_1707	NAC; NAM	34	+	0.97	atACACGgaagt	AT5G66300
TFmatrixID_1708	NAC; NAM	34	+	0.93	atACACGgaagt	AT5G66300
TFmatrixID_1715	NAC; NAM	34	+	0.94	atACACGgaagt	AT1G71930
TFmatrixID_1718	NAC; NAM	34	+	0.94	atACACGgaagt	AT1G71930

TFmatrixID_1719	NAC; NAM	34	+	0.92	atACACGgaagt	AT1G71930
TFmatrixID_1721	NAC; NAM	9	+	0.9	ttccACGGAagt	AT1G71930
TFmatrixID_1721	NAC; NAM	34	+	0.93	atacACGGAagt	AT1G71930
TFmatrixID_1722	NAC; NAM	9	+	0.94	ttccACGGAagta	AT1G71930
TF_motif_seq_0248	(Motif sequence only)	12	+	0.8	CACGG	MYBCOREATCYCB1
TF_motif_seq_0248	(Motif sequence only)	37	+	0.8	CACGG	MYBCOREATCYCB1
TF_motif_seq_0249	(Motif sequence only)	2	-	0.8	CACGA	ABRELATERD1
TF_motif_seq_0249	(Motif sequence only)	12	-	0.8	CACGG	ABRELATERD1
TF_motif_seq_0249	(Motif sequence only)	25	-	0.8	CAGGT	ABRELATERD1
TF_motif_seq_0249	(Motif sequence only)	37	-	0.8	CACGG	ABRELATERD1
TF_motif_seq_0263	(Motif sequence only)	10	+	0.8	TCCAC	SORLIP1AT
TF_motif_seq_0263	(Motif sequence only)	23	+	0.8	GCCAG	SORLIP1AT
TF_motif_seq_0265	(Motif sequence only)	21	+	0.8	AGGCC	SORLIP2AT
TF_motif_seq_0265	(Motif sequence only)	22	-	0.8	GGCCA	SORLIP2AT
TF_motif_seq_0323	(Motif sequence only)	1	+	1	TCACGa	RHERPATEXPA7

Oryza sativa:

Matrix ID	Family	Position	Strand	Similar Score	Hit Sequence	TF ID or Motif name
TF_motif_seq_0086	(Others)	32	-	0.73	aaatacACGGA	X52153
TF_motif_seq_0257	NF-YB	31	+	0.8	CAAAT	Os01g0834400; Os02g0725900; Os05g0463800; Os05g0573500
TF_motif_seq_0266	TCP	21	+	0.75	AGGCC	Os03g0785800; Os04g0194600; Os12g0616400; Os01g0213800; Os08g0544800; Os11g0175700
TF_motif_seq_0266	TCP	21	-	0.75	AGGCC	Os03g0785800; Os04g0194600; Os12g0616400; Os01g0213800; Os08g0544800; Os11g0175700
TF_motif_seq_0266	TCP	22	+	0.75	GGCCA	Os03g0785800; Os04g0194600; Os12g0616400; Os01g0213800; Os08g0544800; Os11g0175700
TF_motif_seq_0266	TCP	22	-	0.75	GGCCA	Os03g0785800; Os04g0194600; Os12g0616400; Os01g0213800; Os08g0544800; Os11g0175700
TF_motif_seq_0266	TCP	27	+	0.75	GGTAC	Os03g0785800; Os04g0194600; Os12g0616400; Os01g0213800; Os08g0544800; Os11g0175700
TF_motif_seq_0266	TCP	27	-	0.75	GGTAC	Os03g0785800; Os04g0194600; Os12g0616400; Os01g0213800; Os08g0544800; Os11g0175700
TF_motif_seq_0508	SBP	25	+	0.75	caGGTACaa	Os01g0292900; Os01g0922600; Os02g0139400; Os02g0174100; Os02g0177300; Os03g0833300; Os04g0551500; Os05g0408200; Os06g0659100; Os06g0663500; Os06g0703500; Os08g0509600; Os08g0513700; Os08g0531600; Os09g0491532; Os09g0507100; Os11g0496600
TF_motif_seq_0259	(Motif sequence only)	11	+	0.8	CCACG	CGACGOSAMY3
TF_motif_seq_0372	(Motif sequence only)	25	-	0.86	caGGTAC	ACGTOSGLUB1
TF_motif_seq_0425	(Motif sequence only)	24	+	0.75	CCAGGtac	SITEIOSPCNA

Table S4. Presence or absence of the 25 bp InDel in the promoter of TraesCS5A02G542800 in cultivars with different awn length.

Genotype	Awns	D993093	S1134493	Assumed *b1*/*B1*	InDel
Bloyka	long	0	0	*b1*	CACGAAATTCCACGGAAGTAGGCCAGGTACAAATACACGGAAGTA
Centinel	long	0	0	*b1*	CACGAAATTCCACGGAAGTAGGCCAGGTACAAATACACGGAAGTA
Glosa	long	0	0	*b1*	CACGAAATTCCACGGAAGTAGGCCAGGTACAAATACACGGAAGTA
Maksat	long	0	0	*b1*	CACGAAATTCCACGGAAGTAGGCCAGGTACAAATACACGGAAGTA
Cornelius	long	0	0	*b1*	CACGAAATTCCACGGAAGTAGGCCAGGTACAAATACACGGAAGTA
Hans	long	0	0	*b1*	CACGAAATTCCACGGAAGTAGGCCAGGTACAAATACACGGAAGTA
Dbdi	long	0	0	*b1*	CACGAAATTCCACGGAAGTAGGCCAGGTACAAATACACGGAAGTA
Mv Lucia	long	0	0	*b1*	CACGAAATTCCACGGAAGTAGGCCAGGTACAAATACACGGAAGTA
Mv-Beres (1)	long	0	0	*b1*	CACGAAATTCCACGGAAGTAGGCCAGGTACAAATACACGGAAGTA
Ekiz	long	0	0	*b1*	CACGAAATTCCACGGAAGTAGGCCAGGTACAAATACACGGAAGTA
Konya	long	0	0	*b1*	CACGAAATTCCACGGAAGTAGGCCAGGTACAAATACACGGAAGTA
MV-Taltos	long	0	0	*b1*	CACGAAATTCCACGGAAGTAGGCCAGGTACAAATACACGGAAGTA
Corsaire	no/short	1	2	*B1*	CACGAAATTC-------------------------CACGGAAGTA
Estica	intermediate	1	2	*B1*	CACGAAATTC-------------------------CACGGAAGTA
Muza	intermediate	1	2	*B1*	CACGAAATTC-------------------------CACGGAAGTA
Aperitiv	no/short	1	2	*B1*	CACGAAATTC-------------------------CACGGAAGTA
Esket	no/short	1	2	*B1*	CACGAAATTC-------------------------CACGGAAGTA
Barryton	no/short	1	2	*B1*	CACGAAATTC-------------------------CACGGAAGTA
Simonida	intermediate	1	2	*B1*	CACGAAATTC-------------------------CACGGAAGTA
Impression	no/short	NA (1 imp)	2	*B1*	CACGAAATTC-------------------------CACGGAAGTA
Batjko	intermediate	1	2	*B1*	CACGAAATTC-------------------------CACGGAAGTA
Event	no/short	1	2	*B1*	CACGAAATTC-------------------------CACGGAAGTA
Novo Zvesda	no/short	1	2	*B1*	CACGAAATTC-------------------------CACGAAAGTA
Stava	no/short	NA (1 imp)	2	*B1*	CACGAAATTC-------------------------CACGGAAGTA
Potenzial	no/short	1	2	*B1*	CACGAAATTC-------------------------CACGGAAGTA
Kranich	no/short	1	2	*B1*	CACGAAATTC-------------------------CACGGAAGTA

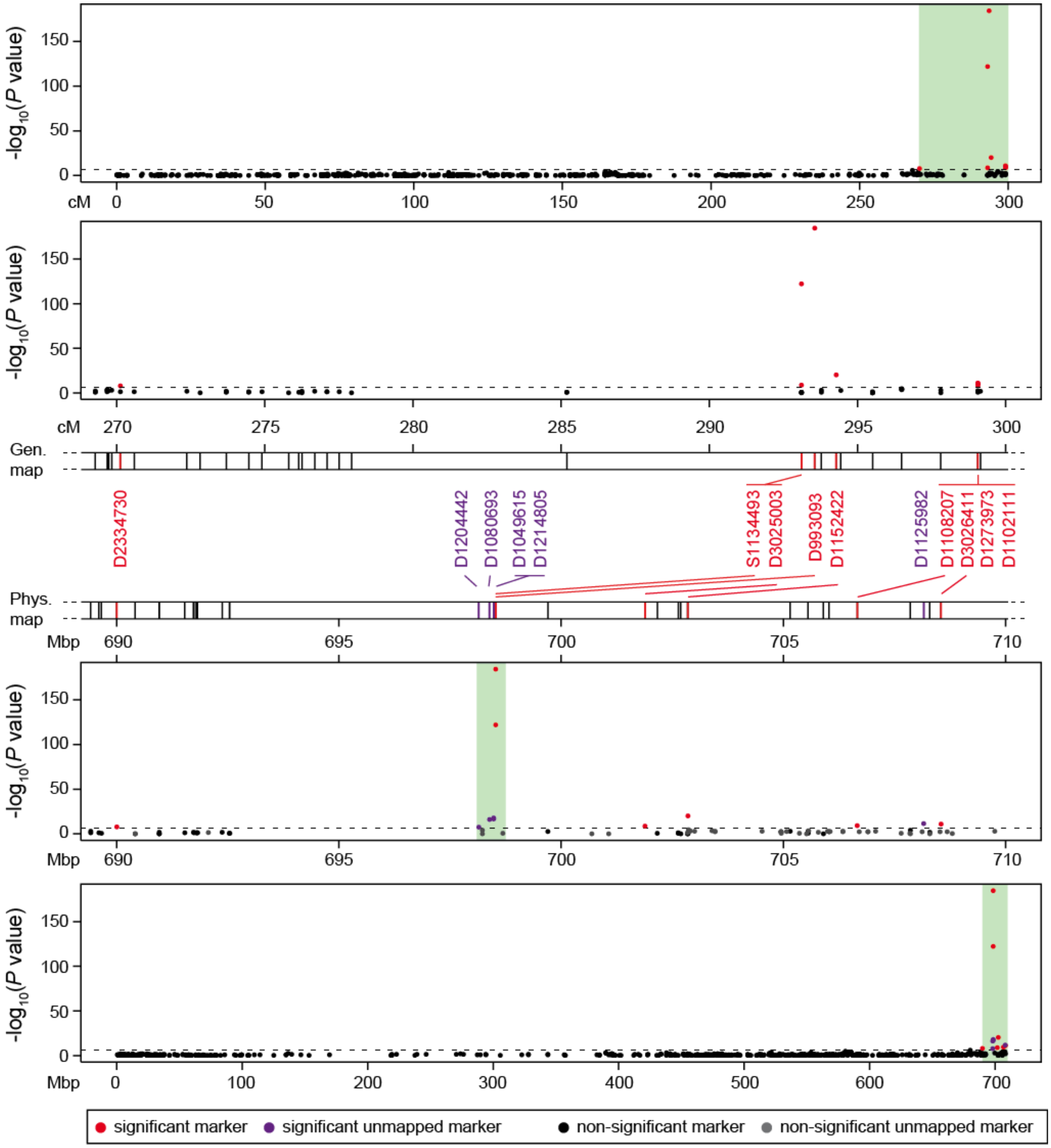

Fig. S1. Genetic and physical fine-mapping of the *B1* (*Tipped 1*) locus on chromosome 5A.

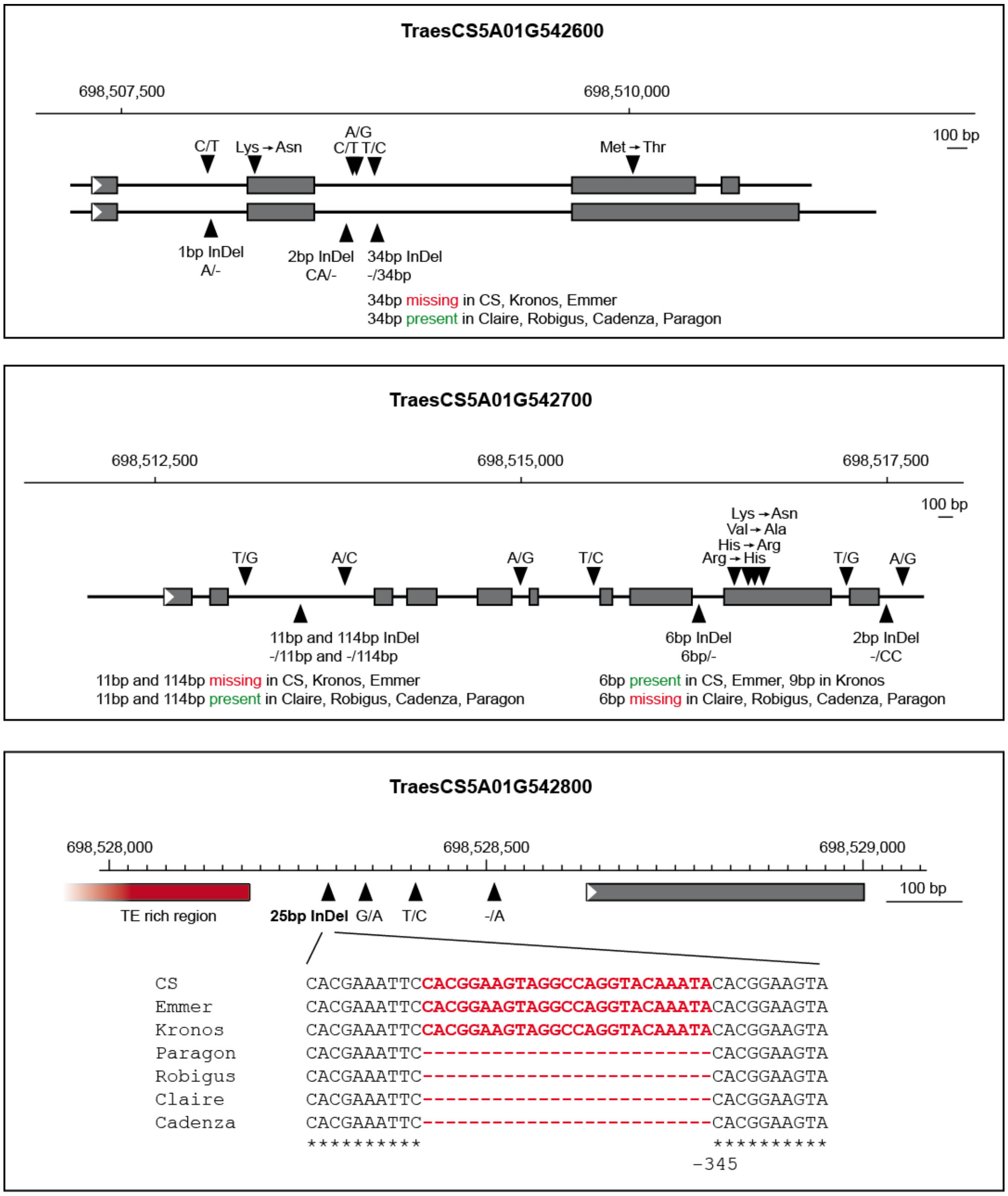

Fig. S2. Polymorphisms in the candidate genes TraesCS5A02G542600, TraesCS5A02G542700 and TraesCS5A02G542800. Polymorphisms are indicated relative to Chinese Spring (CS), i.e. 'T/G' means that CS, wild Emmer and the durum wheat Kronos (all presumed to carry the wild-type *b1* allele) carry a 'T' and Claire, Robigus, Cadenza and Paragon (all presumed to carry *B1*) carry a 'G'. Data were obtained from the Earlham Institute (http://www.earlham.ac.uk/grassroots-genomics) (Avni *et al*., 2017; Bian *et al*., 2017; Clavijo *et al*., 2017).

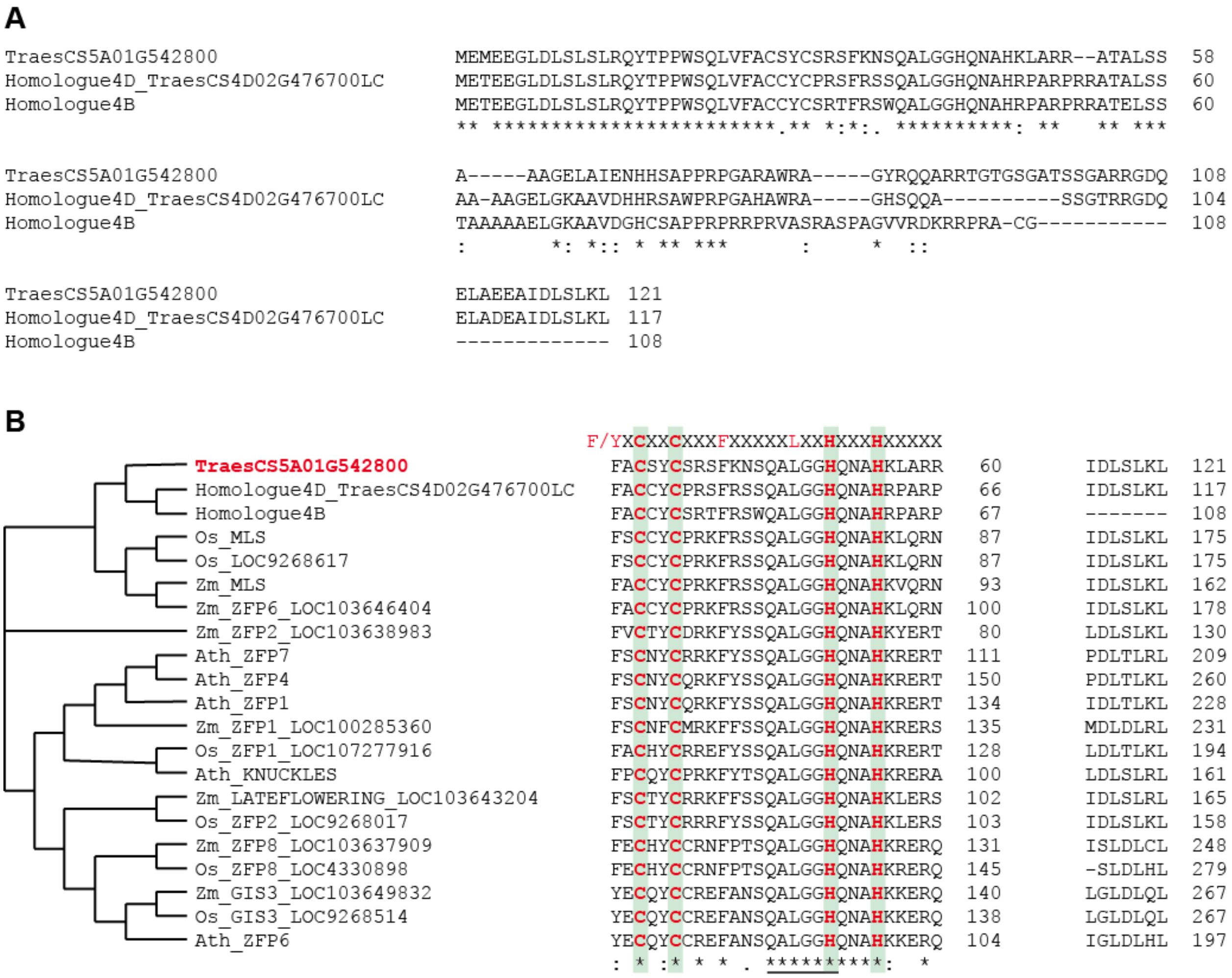

Fig. S3. Alignment of protein sequences by CLUSTAL O(1.2.4). (A) The candidate gene TraesCS5A02G542800 and its two putative homologues; the non-annotated 4B homologue lacking the C-terminal region. (B) Multiple sequence alignment of these three wheat proteins, as well as proteins from *Arabidopsis thaliana* (Ath), *Oryza sativa* (Os) and *Zea mays* (Zm) showing similarity in a BLAST against these species. The zinc-finger domain and the EAR-like motif (defined as LxLxL or DLNxxP; Kagale and Rozwadowski 2011) are shown; the name-giving C2H2 cysteine and histidine residues are highlighted, and the submotif QALGGH present in many plant zinc finger proteins is underlined (Takatsuji 1999).

plants.

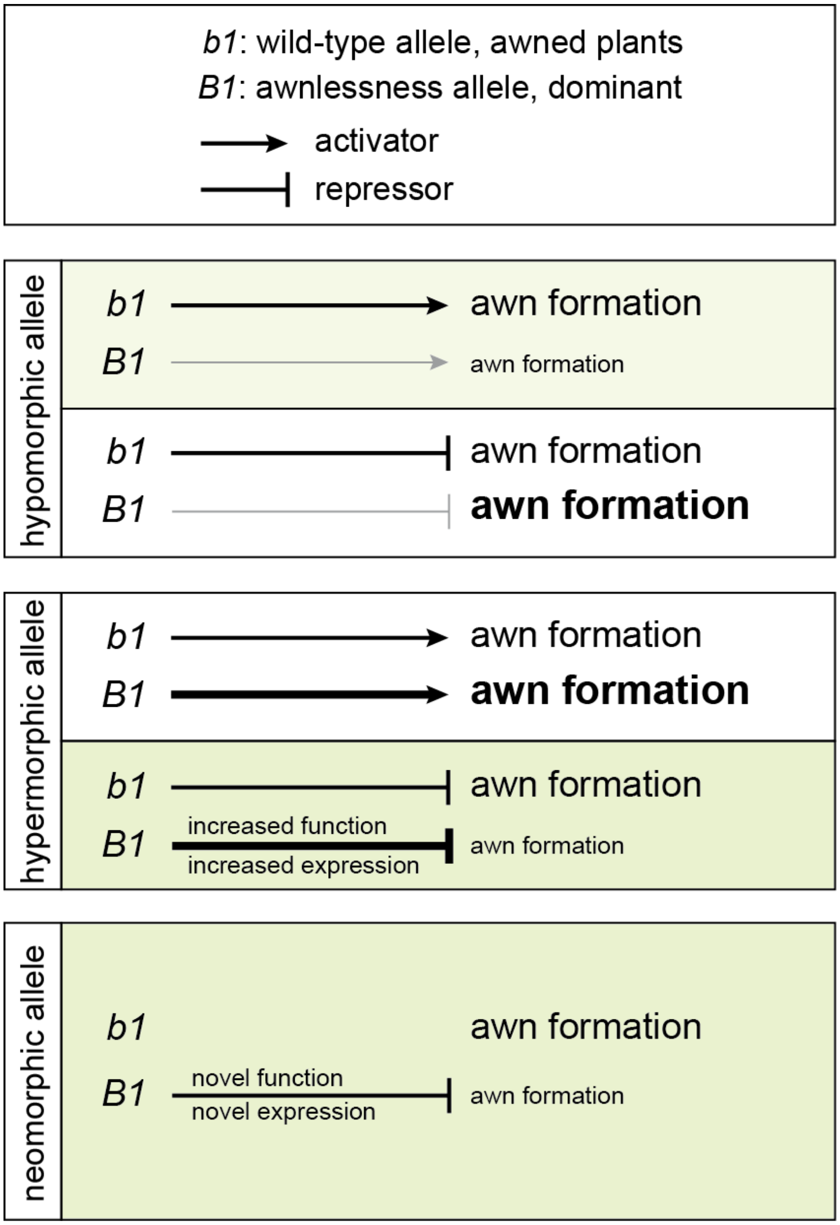

Fig. S4. Schematic presentation of the possible modes of action of the wild-type *b1* allele and the awnlessness-conferring *B1* allele, shown for the theoretical scenarios of *B1* being a hypomorphic (reduced activity), hypermorphic (increased activity), or neomorphic (novel activity) allele. *B1* being a hypomorphic allele of an activator of awn formation appears less likely, as then *B1* could only be dominant if there was a threshold level of activity that is not reached in heterozygous plants.

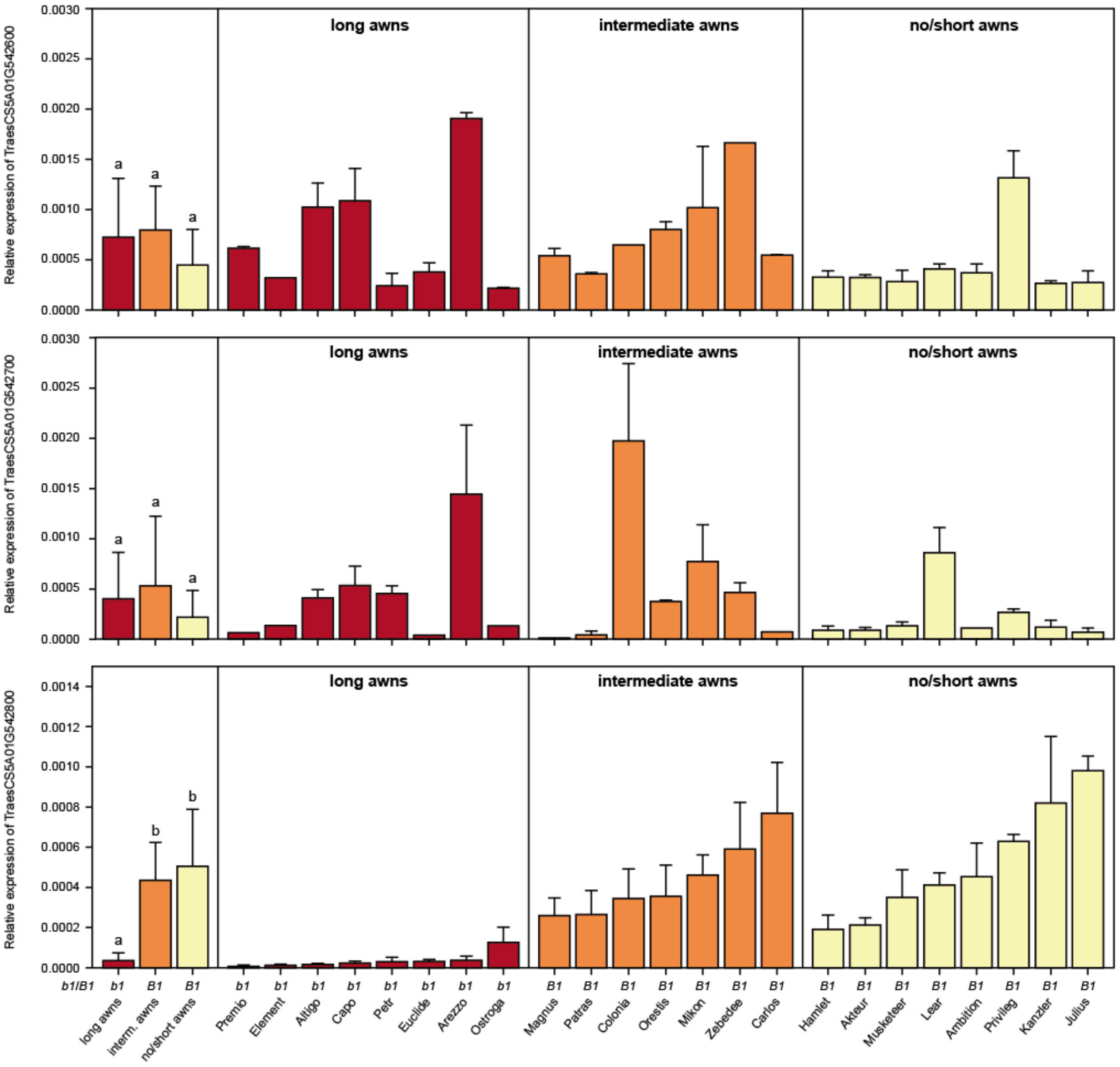

Fig. S5. Expression of the three candidate genes TraesCS5A02G542600, TraesCS5A02G542700 and TraesCS5A02G542800 in developing spikes determined by qPCR. Samples were taken from at least ten plants per genotype. The whiskers represent the standard deviation and different letters indicate significant differences between the three groups.
